# Einstein–Roscoe regression for the slag viscosity prediction problem in steelmaking

**DOI:** 10.1038/s41598-022-10278-w

**Published:** 2022-04-21

**Authors:** Hiroto Saigo, Dukka B. KC, Noritaka Saito

**Affiliations:** 1 Department of Electrical Engineering and Computer Science, Kyushu University, 744, Motooka, Nishi-ku, 819-0395 Japan; 2 Department of Computer Science, Michigan Technological University, Houghton, MI 49931 USA; 3 Department of Materials, Kyushu University, 744, Motooka, Nishi-ku, 819-0395 Japan

**Keywords:** Computer science, Scientific data, Statistics

## Abstract

In classical machine learning, regressors are trained without attempting to gain insight into the mechanism connecting inputs and outputs. Natural sciences, however, are interested in finding a robust interpretable function for the target phenomenon, that can return predictions even outside of the training domains. This paper focuses on viscosity prediction problem in steelmaking, and proposes Einstein–Roscoe regression (ERR), which learns the coefficients of the Einstein–Roscoe equation, and is able to extrapolate to unseen domains. Besides, it is often the case in the natural sciences that some measurements are unavailable or expensive than the others due to physical constraints. To this end, we employ a transfer learning framework based on Gaussian process, which allows us to estimate the regression parameters using the auxiliary measurements available in a reasonable cost. In experiments using the viscosity measurements in high temperature slag suspension system, ERR is compared favorably with various machine learning approaches in interpolation settings, while outperformed all of them in extrapolation settings. Furthermore, after estimating parameters using the auxiliary dataset obtained at room temperature, an increase in accuracy is observed in the high temperature dataset, which corroborates the effectiveness of the proposed approach.

## Introduction

In classical machine learning, regressors are trained with a training dataset, and the generalization performances are measured using a test set. If the dataset at hand is dense enough, then the problem boils down to an *interpolation* problem. However, it is often the case that the measurements are available in a limited domain due to the constraints imposed by the physical environment. In natural sciences, still regressors are desired to return robust and accurate predictions even outside of the training domain, since they can help researchers formulating hypotheses about the observed phenomenon. We call this situation as *extrapolation*, and aim at building robust and accurate regressors both in the interpolation and extrapolation domains.

We are particularly interested in the slag viscosity prediction problem in steelmaking industry. The slag viscosity is known to be a key parameter in controlling and understanding industrial process, but no existing approach can measure it directly in the working blast furnace. To this end, in the real operation scene, slag is usually treated simply as pure liquid. However, the real slag is considered to be a multiphase fluid consisting of solid and liquid^1,2^. Thereby based on this idea, we attempt to model the slag viscosity as a function of the fraction of solid and liquid phases, shape and size of the solid phase particles. The validity of the obtained model is to be verified using our in-house data obtained in the high temperature slag suspension system. In this system, we are able to measure the viscosity using the rotation method (Please refer to “Methods” section for more details). Provided that we could have built an accurate prediction model with our high temperature slag data, then it would support our hypothesis that the slag in high temperature exists not simple in a liquid phase, but in multiphase consisting of liquid and solid.

**Figure 1:**
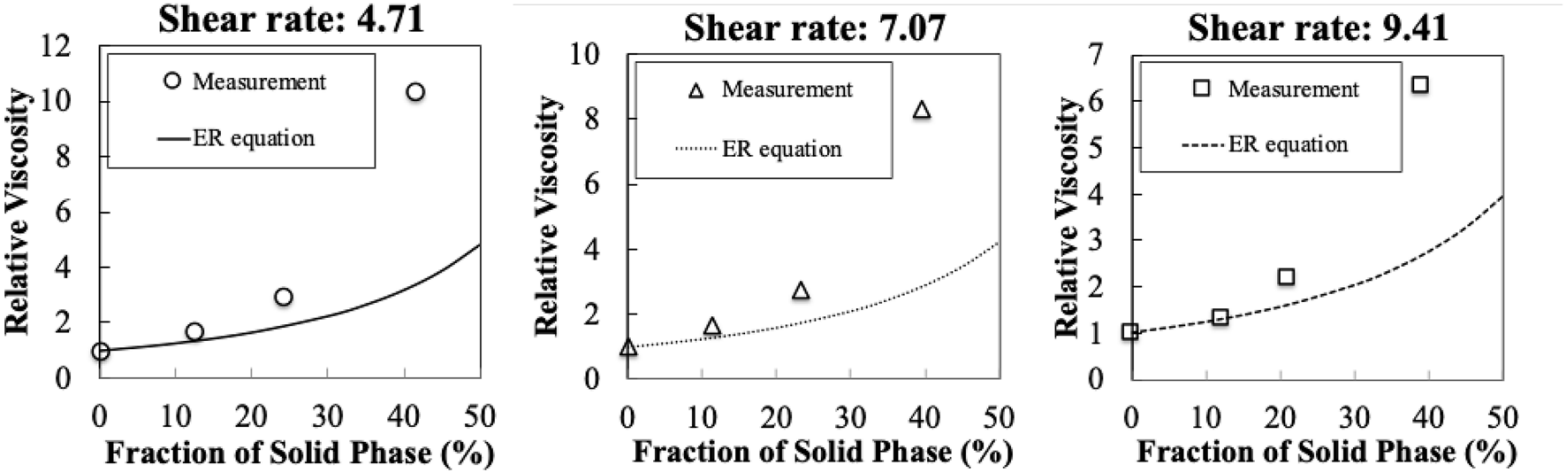
Underestimation results of the viscosity by an Einstein–Roscoe equation. The measurements are obtained in a matrix of ${\mathrm{CaO}}-{\mathrm{Al}}_2{\mathrm{O}}_3-{\mathrm{SiO}}_2-{\mathrm{MgO}}$ slag at 1773 K. Each plot corresponds to a different shear rate. The details regarding the experimental system is described in the “Methods” section.

In the field of chemical engineering, numerous viscosity models including Einstein–Roscoe^3^, Krieger-Dougherty^4^ have been developed. However, there exists no universally valid model primarily due to an over simplification of the model despite the complicated target system^5^, which applies to our case as well. For a specific example, we showcase the fitting of the Einstein–Roscoe equation to our in-house dataset measured in a high temperature (1773 K) experimental system in Fig. 1. It is observed that the fitting of the Einstein–Roscoe equation consistently underestimated the true measurements. In order to overcome this situation, we propose a novel regression algorithms specifically designed for the slag viscosity prediction problem in steelmaking.

In this paper we also address another important problem in experimental science and industry; supplementing the number of high-cost experiments using dataset measured in a reasonable cost. In our specific case, the measurements of viscosity in the high temperature slag suspension system are quite expensive. However, a room temperature experimental system has been designed to mimic the behavior in the high temperature system such that the number of measurements can be compensated^6^. Then we desire to estimate important parameters for the prediction model in the high temperature system using the auxiliary measurements from the room temperature system. The corresponding problem is known as *transfer learning* in machine learning. We employ Gaussian process^7^ for this task, and attempt to estimate its parameters in the direction of maximizing the marginal likelihood using the auxiliary dataset.

We make two main contributions in this paper: (1) proposal of the Einstein–Roscoe Regression (ERR), a model based on the Einstein–Roscoe equation, and (2) the development of the parameter selection strategy using the auxiliary dataset.

This paper is organized as follows. In the next section, we describe the main results including the main contributions. In “Discussion” section, we argue the limitation and the possible extension of the proposed approach. “Methods” section describes the procedures of the computational experiments and the physical experiments.

## Results

In this section, we first illustrate the problem, introduce the proposed model, and evaluate it both in the simulated datasets and the high temperature slag suspension datasets. We then extend the proposed method such that it can make use of the auxiliary dataset measured at room temperature.

### Einstein–Roscoe regression (ERR) in the simulated datasets

For simplicity, we begin by estimating the viscosity solely based on the fraction of the solid phase, and ignore the other parameters for the moment. The simulation dataset is generated based on the Einstein–Roscoe equation^3^, a popular equation for modeling the viscosity of heterogeneous silicate melts;

1 $$\begin{aligned} \eta _r = (1-\phi )^{-n}, \end{aligned}$$

where $\eta _r$ stands for relative viscosity and $\phi$ stands for the fraction of solid phase. *n* is typically determined by the particle size, shear rate, and kinetic viscosity of the liquid phase. In^8^, *n* is determined using the Reynolds number such that $n = 0.362 Re^{-0.189}$, where *Re* is determined by $\frac{(d/2)^2 \gamma \rho }{\eta _L}$, and *d*, $\rho$ and $\eta _L$ are the diameter of the particle, shear rate and the viscosity of the liquid phase, respectively. The coefficients 0.362 and $-0.189$ are obtained by non-linear regression to the measurements^6^. It is based on a system of physical models, and allows us to understand the reason of the viscosity, but did not reproduce the viscosity in the high temperature system as shown in Fig. 1. To this end, we attempt a data science approach; estimate the coefficients *n* of Einstein–Roscoe equation by least squares, that is, we solve

2 $$\begin{aligned} \log (1-\phi ) \; \tilde{n} = -\log (\eta _r), \end{aligned}$$

for $\tilde{n}$. This approach is completely data-driven, and different from any physical model proposed in the literature^5^. We call this approach as Einstein–Roscoe Regression (ERR) below.

**Figure 2:**
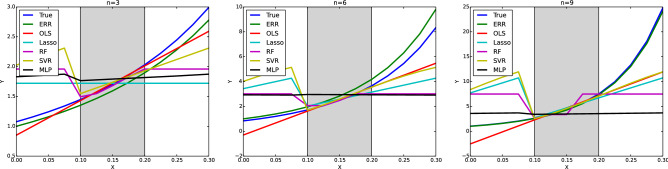
Prediction results of the regressors in the simulated datasets. Each plot corresponds to a different coefficient, and they are $n=3$(left), $n=6$(center), and $n=9$(right). All the regressors except for ERR show poor extrapolation abilities.

The results of fitting ERR and various machine learning algorithms to the simulated datasets are shown in Fig. 2. In this simulation experiment, we first fixed *n*, generated data points according to Einstein–Roscoe equation, and added Gaussian noise. Then we trained ERR and the baseline methods using the data points whose domain are limited to $\phi =\{0.1,0.125,0.15,0.175,0.2\}$ for simulating the extrapolation settings. The gray band in the middle of each figure corresponds to the training domain, and the neighboring left and right domains correspond to the test domains. The baseline methods we tested includes Ordinary Least Squares (OLS), Lasso^9^, Random Forest (RF)^10^, Support Vector Regression (SVR)^11^ and Multi Layer Perceptron (MLP)^12^. It is clear from Fig. 2 that baseline approaches perform well in *interpolation* settings, but are quite erroneous in *extrapolation* settings. Our proposed method ERR, on the other hand, correctly captures the *smooth* and *nondecreasing* properties of the viscosity with respect to the solid phase rate. The resulting good performance in both the *interpolation* and *extrapolation* problems is due to the usage of the Einstein–Roscoe equation as a prior knowledge.

**Figure 3:**
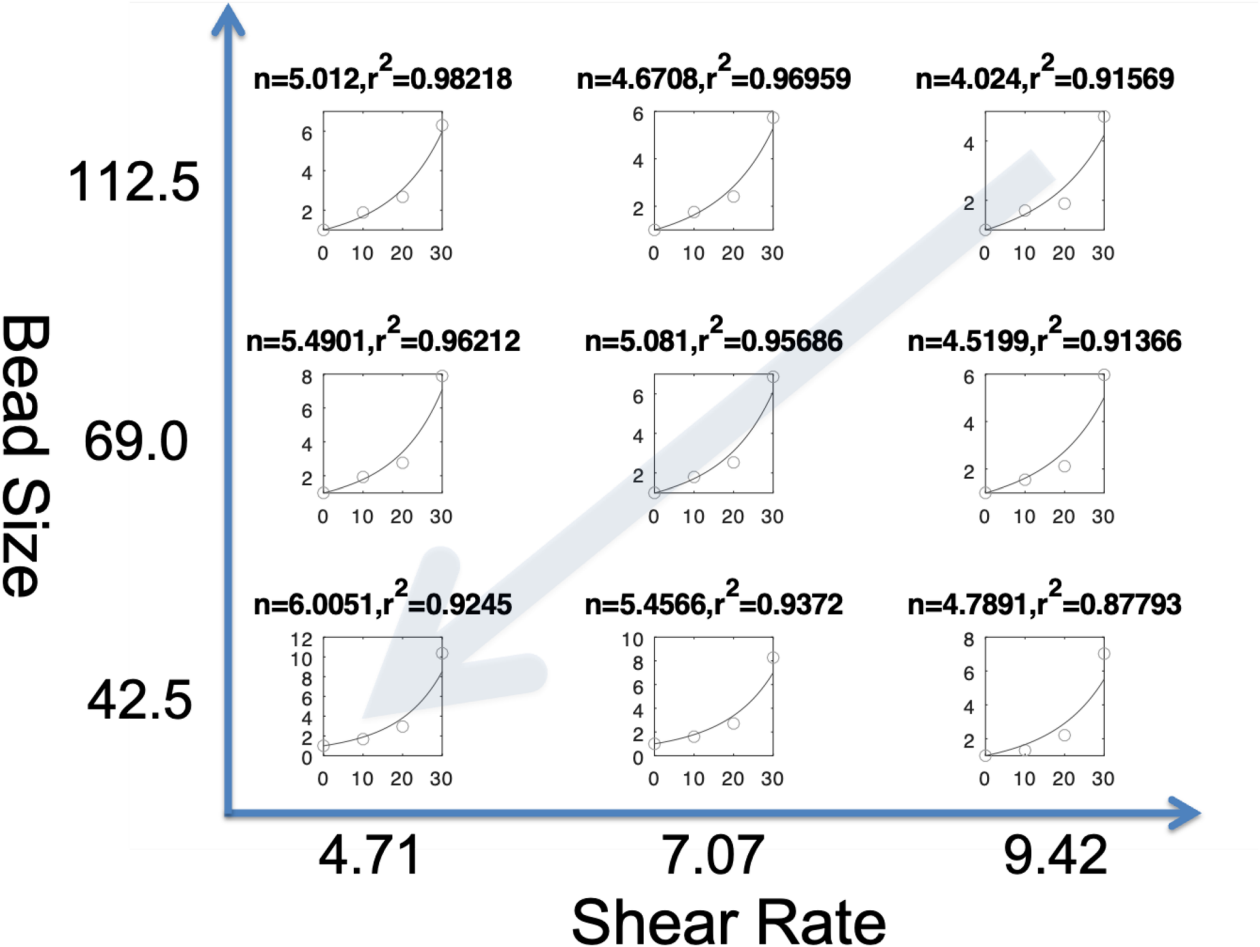
Least squares fitting by ERR to the high temperature slag suspension data. In each subplot, *x* and *y* axes correspond to the fraction of solid phase rate (%) and the viscosity, respectively. The fitting performance in terms of $r^2$ was as high as between 0.88 and 0.98 across different bead sizes and shear rates. Notice that there are linear relationships (shown as an arrow) between *n* and the bead size, and between *n* and the shear rate.

### Fitting ERR to the high temperature slag suspension data

We demonstrate the effectiveness of the proposed approach using the dataset measured in high temperature slag suspension system. The procedure for the measurements are described in “Methods” section. Figure 3 shows the results of fitting ERR to the measurements across different shear rates and bead (particle) sizes. The resulting $r^2$ was between 0.878 and 0.982, indicating the good fitting performance. Moreover, we have two interesting observations from the estimated coefficients *n*; (1) *n* increases with the decreasing bead size, and (2) *n* increases with the decreasing shear rate. Both facts are consistent with the previous observation^6^. We can see in Fig. 3 that we have obtained 9 estimates of the coefficient *n* of Einstein–Roscoe equation. In our ERR framework below, we use these estimated coefficients *n* as true response label, and attempt to estimate them from the other experimental parameters such that;

3 $$\begin{aligned} n=f(d,\gamma ), \end{aligned}$$

where *d* denotes bead size, and $\gamma$ denotes shear rate. Notice that once *n* is determined, then the viscosity $\eta _r$ can be obtained by Eq. (1).

### Extrapolation experiments using the high temperature slag suspension data

**Figure 4:**
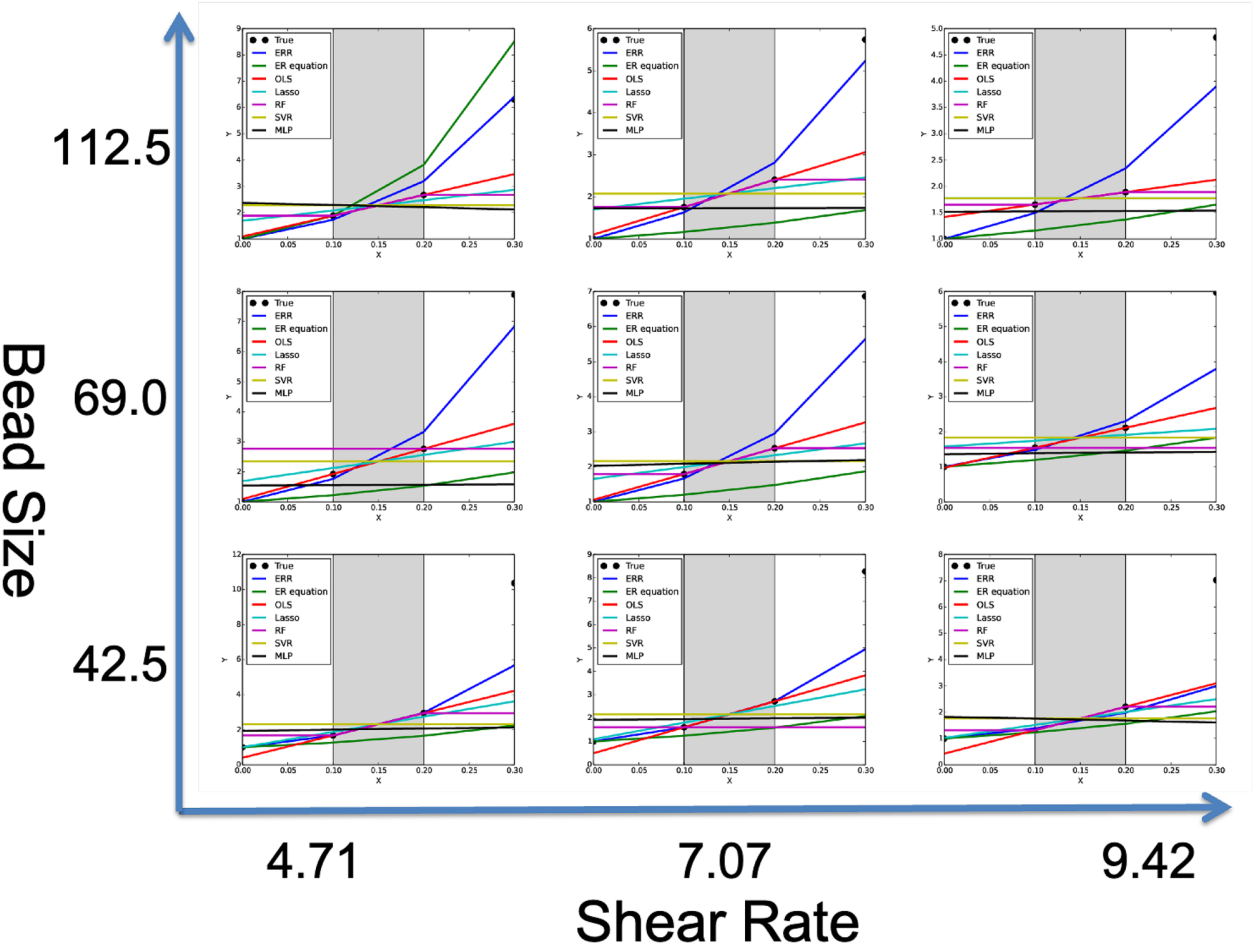
The prediction results in different shear rates and bead sizes. The measurements in the gray band are used for training, and the measurements in the neighboring regions are used only for the testing. The ER equation underestimated the viscosity for most of the cases. All the other baseline machine learning methods returned flat prediction that goes through the training data points. The proposed method ERR, on the other hand, correctly reproduced the smooth and nondecreasing characteristic due to the usage of Einstein–Roscoe model as a prior knowledge.

We investigate the effectiveness of ERR compared with the baseline ER equation and various machine learning algorithms in Fig. 4. The experimentally measured data points are denoted as True (black circles), and the prediction result of each method is displayed in a line. In this dataset, we have only four data points along the *x* axis; $x=\{0, 0.1, 0.2, 0.3\}$, but we have reserved 0 and 0.3 for the extrapolation settings, and used only 0.1 and 0.2 for training. The resulting problem is quite hard, since we have only two points for estimating a non-linear curve.

**Table 1:** Prediction errors in terms of mean squares in both interpolation and extrapolation settings in the high temperature viscosity prediction problem.

Domain	ERR	ER equation	OLS	LASSO	RF	SVR	MLP
Interpol. (train)	0.075 ± 0.065	0.60 ± 0.29	0.00 ± 0.00	0.037 ± 0.0087	0.13 ± 0.22	0.18 ± 0.12	0.32 ± 0.19
Extrapol. (test)	**3.2** ± **4.1**	13 ± 9.2	301 ± 630	10 ± 5.5	12 ± 7.0	14 ± 8.6	15 ± 8.7

Statistically significant results are highlighted in a bold font.

As expected, all the existing regressors performed well inside of the training domain, but poorly outside (See Fig. 4). The ER equation underestimated the viscosity for most of the situations. In contrast to that, our proposed method ERR performed well in both interpolation and extrapolation settings. In order to make a detailed comparison, we summarized the errors in terms of mean squares of each regressor in Table 1. In the interpolation settlings, there was no statistically significant difference by a one-sided two sample t-test with 5% significance level (except for OLS whose fitting line passes directly on the training data points, resulting in zero error). In the extrapolation settings, the differences between ERR and all the others were statistically significant by a one-sided two sample t-test with 5% significance level (highlighted in a bold font).

### Extension of the basis model and the estimation of model parameters using the auxiliary data

Thus far, we had only 9 data points for the prediction of the coefficient *n* in the high temperature slag suspension dataset due to the high cost required for the measurements. On the other hand, we have established a room temperature experimental system using polyethylene beads and silicon oil^6^. With this system, we have collected 80 auxiliary data points, and attempt to improve the prediction performance in the high temperature dataset by correctly estimating the common underlying parameters. First, we consider employing a flexible basis model to ERR. We consider *n* as a function of $\gamma$ and *d*, and employ Gaussian process^7^. As a result the prediction model can be represented as a weighted sum of kernels.

4 $$\begin{aligned} n(x)=\sum _{i} \alpha _i k(x_i,x), \end{aligned}$$

where *x* corresponds to the measurements with different $\gamma$, *d* and, *i* runs through the number of measurements, and $\alpha$ represents the weights learned by Gaussian process. An advantage of Gaussian process is its ability to tune parameters using the training dataset only. Since we already know that a linear model was successful in the previous experiment (Fig. 3), we consider mixing a linear kernel with a Gaussian kernel^13^ such that an overall trend is captured by a linear model, and small fluctuations are captured by a Gaussian model. The procedure for this experiment is as follows. First we have split the room temperature data into training sets and test sets. Then we have estimated parameters ${\varvec{\theta }}$ in the direction of maximizing marginal likelihood using the conjugate gradient descent. Finally we have run leave-one-out cross-validation in the high temperature dataset using the learned parameters. In oder to highlight the effectiveness of the parameter selection, we compare the kernel before and after optimizing parameters. A kernel before the parameter optimization is given in the following form;

5 $$\begin{aligned} k_{Customized}({{x}},{{x}}'\mid {{\theta }}) = \langle {{x}},{{x}}'\rangle + \exp \left( -|{{x}}-{{x}}'|^2\right) +\delta ({{x}},{{x}}'). \end{aligned}$$

We also compare a kernel with only a linear term;

6 $$\begin{aligned} k_{Linear}({{x}},{{x}}'\mid {{\theta }}) = \langle {{x}},{{x}}'\rangle . \end{aligned}$$

**Figure 5:**
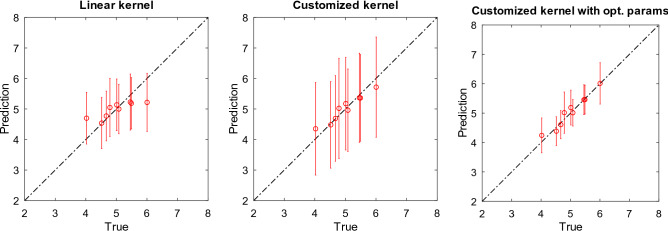
Prediction results of the coefficient *n* of Einstein–Roscoe equation. A plot with circles lying closer to the diagonal represents better prediction performance, and the predictions with smaller prediction intervals (vertical bar) represents higher confidence in the prediction. Customized kernel with optimized parameters (right most) not only achieved the best prediction performance, but displayed the higher confidence in the prediction than the other kernels.

In Table 2, we compare different kernels in terms of root mean squared errors. The errors are measured by leave-one-out cross-validation in the high temperature dataset. We can observe that the amount of errors in the optimized kernels have decreased to almost half after parameter selection using the auxiliary dataset. The results of predicted *n* are plotted in Fig. 5, where we can confirm that the predicted points after the parameter estimation lie closer to the diagonal, indicating improved correlation with the true label. We can also observe the smaller prediction intervals after parameter selection, which corresponds to the increase of the confidence in the prediction. Our kernel with optimized parameters are as follows;

7 $$\begin{aligned} k_{Optimized}({{x}},{{x}}'\mid {{\theta }}) = 4.90 \langle {{x}},{{x}}'\rangle + 0.105 \exp \left( -\frac{|{{x}}-{{x}}'|^2}{0.99}\right) +2.92\delta ({{x}},{{x}}'). \end{aligned}$$

A large coefficient to the linear term (4.90) relative to the non-linear model (0.105) indicates inherent linearity over non-linearity. Also a large coefficient to the noise term (2.92) suggests the existence of a relatively large noise in the measurements.

**Table 2:** Errors in terms of mean squares by different kernels in the high temperature dataset.

Kernel type	Linear	Customized	Customized with opt. parameters
MSE	0.147	0.0347	0.0176

## Discussion

A strong linearity in viscosity with respect to the solid phase rate is observed in the two experiments; (1) fitting of ERR to the high temperature dataset and (2) optimization of the kernel parameters. However, an improved prediction performance is obtained by consideration of nonlinear effect into the model, which confirm the validness in the choice of our kernels. In order to achieve robust regression model, we estimated the coefficient *n* of Einstein–Roscoe regression through Eq. (3). It worked successfully in our case at the cost of decreasing the number of data points in high temperature experiments. In practice, we obtained 9 coefficient *n*s, at the cost of the 36 measurements. Generally in machine learning and statistics, a better prediction model comes with more measurements, so one of the future direction would be building a robust and accurate prediction model without decreasing the measurements towards improved prediction performance.

We employed Gaussian process as a regressor to predict the coefficient *n* of the Einstein–Roscoe model. In practice, we can employ any nonlinear regressor such as Multiple Layer Perceptron or Random Forest instead of Gaussian process. However, the properties of the Gaussian process such as the parameter selection ability using the training dataset and the availability of the prediction interval, are unique, and we have successfully made full advantage of them in the experiments.

## Methods

### Einstein–Roscoe regression (ERR)

**Figure 6:**
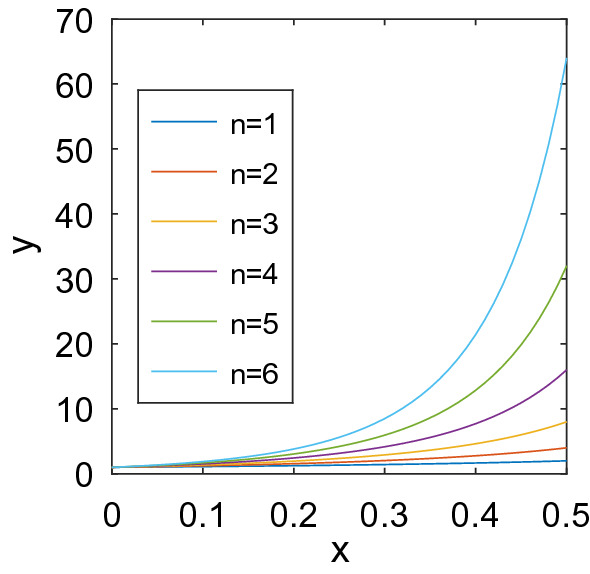
Einstein–Roscoe equation for $a=1$, and various *n*.

Einstein–Roscoe^3^, Krieger-Dougherty^4^ and many other models can be described in the common form as;

8 $$\begin{aligned} \eta _r = (1-a\phi )^{-n}, \end{aligned}$$

where $\phi$ is the fraction of the solid phase particle, and *a* and *n* have been calculated from the shape and size of the solid phase particle in various ways^5^. In this study, we fix $a=1$, and aim at estimating *n* from the training dataset, since *n* is the most important factor that determines the shape of the curve. Figure 6 illustrates that ER equation with various *n* is flexible enough to represent various curves with *smooth* and *nondecreasing* property. Thus we fix our basis model to Eq. (8), and aim at predicting the coefficient *n* from the available measurements.

### Gaussian process and the kernel parameter estimation

Suppose that we are given *d* dimensional feature ${{x}}\in {\mathcal {R}}^d$ and the corresponding response $y\in {\mathcal {R}}$, then the training dataset with *n* examples is represented as ${({{x}}_1,y_1),({{x}}_2,y_2),\ldots ({{x}}_n,y_n)}$. Suppose also that our test set is given as $x_*$, and we aim at predicting the function outputs $f(x_*)$. Let our *kernel function* be *k*(., .), and the element of the kernel matrix computed from the training dataset be ${{K}}_{i,j}=k({{x}}_i,{{x}}_j)$, and those computed using the training dataset and test dataset be $({{k}}_*)_i = k(x_*, x_i)$. The predictive distribution of Gaussian Process is given as

$$\begin{aligned} p(f(x_*)\mid {{X}},{{y}},x_*)={\mathcal {N}}(f(x_*)\mid \mu _*,{{\Sigma }}_*), \end{aligned}$$

where the mean and the covariance of the Gaussian distribution is given as ${{\mu }}_*={{k}}_*^{\top }{{K}}^{-1}{{y}}$ and ${{\Sigma }}_*=k(x_*,x_*)-{{k}}_*^{\top }{{K}}^{-1}{{k}}_*$, respectively. As a kernel function, we employ a mixture of linear and Gaussian kernels in the following form;

9 $$\begin{aligned} k({{x}},{{x}}'\mid {{\theta }}) = \theta _1\langle {{x}},{{x}}'\rangle + \theta _2 \exp \left( -\frac{|{{x}}-{{x}}'|^2}{\theta _3}\right) +\theta _4\delta ({{x}},{{x}}'). \end{aligned}$$

Our high temperature dataset is as small as 9 data points, so we consider using the room temperature dataset for the estimation of the parameters. Since the objective function of the Gaussian process is given as log marginal likelihood

$$\begin{aligned} \log p({{y}}\mid {{\theta }}) = -\frac{1}{2}\log |{{K}}|-\frac{1}{2}{{y}}^{\top }{{K}}^{-1}{{y}}-\frac{n}{2}\log (2\pi ), \end{aligned}$$

we consider taking the gradient with respect to its parameters;

$$\begin{aligned} \frac{\partial {\log p({{y}}\mid \theta )}}{\partial \theta }=-\frac{1}{2}{{\,\mathrm{Tr}\,}}\left( {{K}}^{-1}\frac{\partial {{K}}}{\partial \theta }\right) -\frac{1}{2}{{y}}^{\top }{{K}}^{-1}\frac{\partial {{K}}}{\partial \theta }{{K}}^{-1}{{y}}\end{aligned}$$

With our kernel represented by Eq. (9), we can assume that all the parameters are *nonnegative*. Such constraints can be incorporated by rewriting the parameters as $\theta _i'=e^{\theta _i}$. Then our kernel can be rewritten as

10 $$\begin{aligned} k({{x}},{{x}}'\mid {{\theta }}) = e^{\theta _1'}\langle {{x}},{{x}}'\rangle + e^{\theta _2'} \exp \left( -\frac{|{{x}}-{{x}}'|^2}{e^{\theta _3'}}\right) +e^{\theta _4'}\delta ({{x}},{{x}}'). \end{aligned}$$

Then the gradient with respect to each parameter can be obtained as follows.

$$\begin{aligned} {\left\{ \begin{array}{ll} \frac{\partial k({{x}}_n,{{x}}_n')}{\partial \theta _1'} &= e^{\theta _1'}\langle {{x}},{{x}}'\rangle \\ \frac{\partial k({{x}}_n,{{x}}_n')}{\partial \theta _2'} &= e^{\theta _2'}\exp \left( -\frac{|{{x}}_n-{{x}}_n'|^2}{e^{\theta _3'}}\right) =k({{x}}_n,{{x}}_n')-e^{\theta _4'}\delta (n,n')\\ \frac{\partial k({{x}}_n,{{x}}_n')}{\partial \theta _3'} &= e^{\theta _2'}\exp \left( -\frac{|{{x}}_n-{{x}}_n'|^2}{e^{\theta _3'}}\right) \cdot \frac{\partial }{\partial \theta _3'}\left( -\frac{|{{x}}_n-{{x}}_n'|^2}{e^{\theta _3'}}\right) =(k({{x}}_n,{{x}}_n')-e^{\theta _4'}\delta (n,n'))\cdot e^{-\theta _3'}|{{x}}_n-{{x}}_n'|^2 \\ \frac{\partial k({{x}}_n,{{x}}_n')}{\partial \theta _4}' &= e^{\theta _4'}\delta (n,n') \end{array}\right. } \end{aligned}$$

Given the gradient, we can employ any gradient based optimization method to update the parameters such that;

$$\begin{aligned} \theta \leftarrow \theta +\varepsilon \frac{\partial {\log p({{y}}\mid \theta )}}{\partial \theta }. \end{aligned}$$

In this work, we employed Conjugate Gradient descent optimizer^14^.

### Experimental settings of baseline methods

In this subsection, we describe the parameters used for the baseline methods. The coefficients of Einstein–Roscoe (ER) equation is obtained based on the experimental condition as described in “Results” section. The model is fully described, and there is no parameter to tune.

The baseline machine learning methods has more than or equal to 1 parameter to tune, which is found by cross validation with grid search in the training dataset. In Random Forest (RF), we chose the number of trees from {1,10,100,1000}. In Lasso, regularization parameter is chose from {0.01,0.1,1,10,100}. In SVR, regularization parameter is chose from {0.01,0.1,10,100}, tube size $\epsilon$ is chosen from {0.01,0.05,1.0,1.5,2}, kernel is chosen from either ’linear’ or ’RBF’, and the width parameter of RBF kernel is chosen from {0.01,0.1,1,10,100}. In MLP, the number of hidden layers is chosen from {1,2,3}, and the number of nodes are chosen from {1,3,5,10,20,30}, and the regularization parameter is chosen from {0.0001,0.05}. The max iteration is set to 100, and the SGD optimizer is employed.

### Viscosity measurement of suspensions at room temperature

The viscosity measurement system consists of a rotational viscometer (DVII+ or DV2T, AMETEK Brookfield) and a suspension in a 300 ml beaker. Apparent viscosities were systematically measured for suspensions with different bead volume fractions, average diameters, shear rates, and liquid matrix viscosities. The shear rate was calculated from the rotational speed and the dimensions of the beaker (inner diameter 73 mm) and spindle (outer diameter 3.2 mm) using the following equation

$$\begin{aligned} \gamma =\frac{2\omega }{1-(r_i/r_o)^2} \end{aligned}$$

where $\gamma ,\omega ,r_o,r_i$ are the shear rate, angular velocity, inner radius of the outer cylinder (beaker), and radius of the inner cylinder (spindle), respectively. The apparent viscosity of the suspension was calculated from the torque generated in the spindle by rotation. The variation of the apparent viscosity was about 10%. Silicone oil (KF-96, Shin-Etsu Chemical) with viscosities of 0.5, 1.0, 2.0, and 3.0 Pa-s at 24 $^{\circ }$C was used as the low polarity liquid matrix, and the relative permittivity ranged from 2.7 to 2.8, depending on the viscosity. Polyethylene beads (LE-1080, Sumitomo Seika) with average diameters of 9.35, 162.5, 340.0, and 602.5 $\mu m$ were selected as dispersed solid particles, whose sphericity and particle distribution were confirmed to be relevant by using scanning electron microscopy (SEM) imaging. Please refer to^6^ for more details.

### Viscosity measurement of slag suspensions at high temperature

Calcined CaO and MgO powders were added to CaO and MgO saturated slag, respectively, to prepare a suspension of CaO and MgO particles dispersed in the slag. According to the phase diagram, the chemical composition of a quasi-ternary system 53CaO–35Al2O3–3SiO2-8MgO (mass %), has the eutectic temperature of lime (CaO) and periclase (MgO) at 1773 K, suggesting that CaO and MgO do not chemically dissolve at the selected composition and at 1773 K. As reagents, powders of CaCO3, Al2O3, SiO2, and MgO (Sigma-Aldrich Japan) were carefully weighed to achieve the specified composition and thoroughly mixed in an alumina mortar. The powder batches were pre-melted in a resistance furnace using a platinum crucible for 1 h at 1873 K in air and quenched on a water-cooled copper plate. The respective reagent powders were calcined at 1473 K for 30 min to prepare CaO and MgO particles for dispersion. The calcined and sieved CaO and MgO particles were mixed with a pre-melted 53CaO–35Al2O3–3SiO2–8MgO (mass%) slag to a predetermined composition before the viscosity measurement. Viscosity measurements were performed using the rotating crucible viscometer apparatus as described in the previous section. A Pt-20mass%Rh crucible filled with the mixed slag and CaO or MgO particles was placed in the crucible supporter in the furnace, heated to 1773 K, and then the viscosity of the CaO or MgO slag dispersion was measured at the same temperature. Please refer to^6^ for more details.
